# YTHDF2 suppresses the plasmablast genetic program and promotes germinal center formation

**DOI:** 10.1016/j.celrep.2022.110778

**Published:** 2022-05-06

**Authors:** Amalie Grenov, Hadas Hezroni, Lior Lasman, Jacob H. Hanna, Ziv Shulman

**Affiliations:** 1 Department of Systems Immunology, Weizmann Institute of Science, Rehovot, Israel; 2 Department of Molecular Genetics, Weizmann Institute of Science, Rehovot, Israel

**Keywords:** germinal center, B cells, antibodies, YTHDF, plasma cells, m6A, mRNA, epigenetics, post-transcriptional

## Abstract

Antibody-mediated immunity is initiated by B cell differentiation into multiple cell subsets, including plasmablast, memory, and germinal center (GC) cells. B cell differentiation trajectories are determined by transcription factors, yet very few mechanisms that specifically determine early B cell fates have been described. Here, we report a post-transcriptional mechanism that suppresses the plasmablast genetic program and promotes GC B cell fate commitment. Single-cell RNA-sequencing analysis reveals that antigen-specific B cell precursors at the pre-GC stage upregulate YTHDF2, which enhances the decay of methylated transcripts. Ythdf2-deficient B cells exhibit intact proliferation and activation, whereas differentiation into GC B cells is blocked. Mechanistically, B cells require YTHDF2 to attenuate the plasmablast genetic program during GC seeding, and transcripts of key plasmablast-regulating genes are methylated and bound by YTHDF2. Collectively, this study reveals how post-transcriptional suppression of gene expression directs appropriate B cell fate commitment during initiation of the adaptive immune response.

## Highlights

• scRNA-seq of antigen-specific B cells reveals differentiation trajectories
• YTHDF2 is expressed by early-responding B cells and facilitates germinal center seeding
• YTHDF2 binds mRNAs of plasma cell-associated genes and suppresses their expression
• Germinal center formation does not depend on YTHDF1 and YTHDF3

## Graphical abstract

**Figure:**
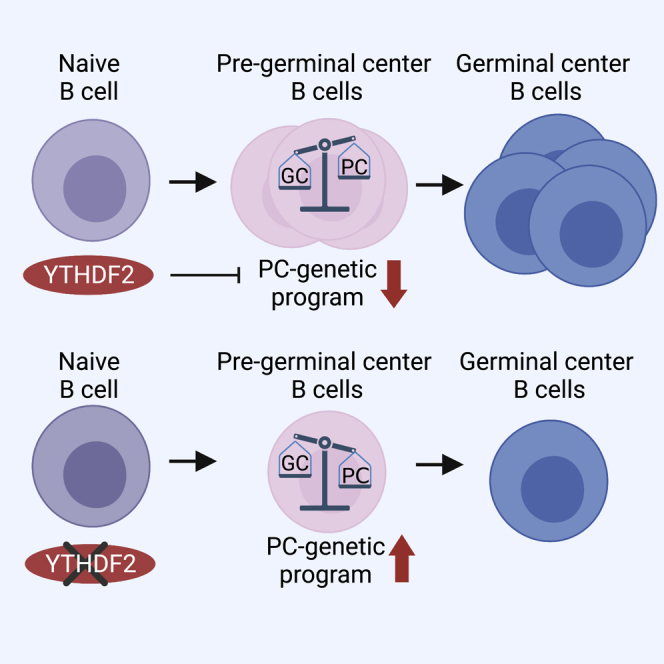

## Teaser

Using scRNA-seq and transgenic mice, Grenov et al. demonstrate that YTHDF2 is expressed by early-responding antigen-specific B cells and promotes their differentiation into germinal center B cells through the repression of genes associated with plasma cell formation, while other YTHDF paralogs are dispensable for the humoral immune response.

## Introduction

The adaptive immune response has the capacity to generate protective antibodies and long-lasting immunological memory, which provides a rapid response to recurrent pathogen exposures (Ahmed and Gray, 1996; Tarlinton and Good-Jacobson 2013; Cyster and Allen, 2019). Activation of naive B cells with cognate antigen combined with signals from T follicular helper (Tfh) cells leads to the development of plasmablasts (PBs), memory, and germinal center (GC) B cells (Shlomchik and Weisel, 2012). Typically, the first wave of antibodies elicited against an invading pathogen consists of germline-encoded class-switched and unswitched immunoglobulins produced by short-lived antibody-secreting cells (Elsner and Shlomchik, 2020). Long-lived immunoglobulin M (IgM) and class-switched memory cells also arise at the early stages of the response (Good-Jacobson and Tarlinton, 2012; Taylor et al., 2012; Weisel et al., 2016; Pritchard and Pepper, 2018). In parallel, GC B cells differentiate from rapidly proliferating B cell subsets and form cell clusters in the center of the lymph node follicle 5–7 days after exposure to immune stimulation. The GC structures persist for several weeks to months, depending on the nature of the pathogen or vaccination, and generate long-lived memory and plasma cells (PCs) that typically carry mutated high-affinity immunoglobulins (Victora and Nussenzweig, 2012).

Regulation of the developmental dichotomy between GC B cells and antibody-secreting cells (ASCs, defined as either PBs or PCs) has been extensively studied (Oracki et al., 2010; Tellier and Nutt, 2019). Differentiation of antigen-specific B cells that encountered a cognate antigen to plasma and GC cells, and stabilization of alternative gene programs are dependent on the silencing of reciprocal fate-inducing transcripts, which is mediated by BLIMP1 and BCL6 (Nutt et al., 2011). IRF4 has a dual role in B cell fate commitment as it promotes both the formation of PBs as well as GC B cell fate (Klein et al., 2006). In a model known as “kinetic control” (Ochiai et al., 2013), it was suggested that intermediate levels of IRF4 promote B cell differentiation into GC B cells, whereas sustained high expression levels promote the formation of PCs through enhanced transcription of PC-related genes, including *Prdm1*, encoding BLIMP1 (Cook et al., 2020).

Post-transcriptional events and RNA-binding proteins (RBPs) such as hnRNPLL, PTBP1, Hur, and ELAV1 play key roles in supporting B cell activation and GC formation (Diaz-Muñoz et al., 2015; Díaz-MonzónCasanova et al., 2018; Chang et al., 2015; Díaz-Muñoz and Turner, 2018). Most of these factors regulate essential biological processes in B cells, including cell cycle, cell metabolism, and RNA splicing rather than specific cell fate decisions (Díaz-Muñoz and Turner, 2018). During transcription, nascent mRNAs are modified by a methyltransferase, which adds a methyl group on adenosines (m^6^A) at consensus sites (Shulman and Stern-Ginossar, 2020). The YTHDF protein family consists of three paralogs, all of which harbor a YTH motif that directly interacts with methylated mRNAs (Zaccara et al., 2019). Although several functions were suggested, the primary role of YTHDFs is to suppress gene expression by enhancing the degradation of methylated mRNA in the cytoplasm (Ivanova et al., 2017; Zaccara et al., 2019). This mechanism supports several essential biological processes in B cells (Zheng et al., 2020; Grenov et al., 2021), but whether it has a role in cell fate commitment has remained unclear. Furthermore, a comprehensive investigation of the functions of each YTHDF paralog in the immune system has not been conducted.

Here, using single-cell RNA sequencing (scRNA-seq) and transgenic mice, we found that YTHDF2 suppresses the PB genetic program and promotes GC formation during the early phases of the B cell immune response. Other YTHDF paralogs, which are expressed at lower levels, mildly modulate B cell gene programs and do not show functional redundancy with YTHDF2.

## Results

### YTHDF2 is expressed by antigen-specific B cells at the pre-GC stage

**Figure 1:**
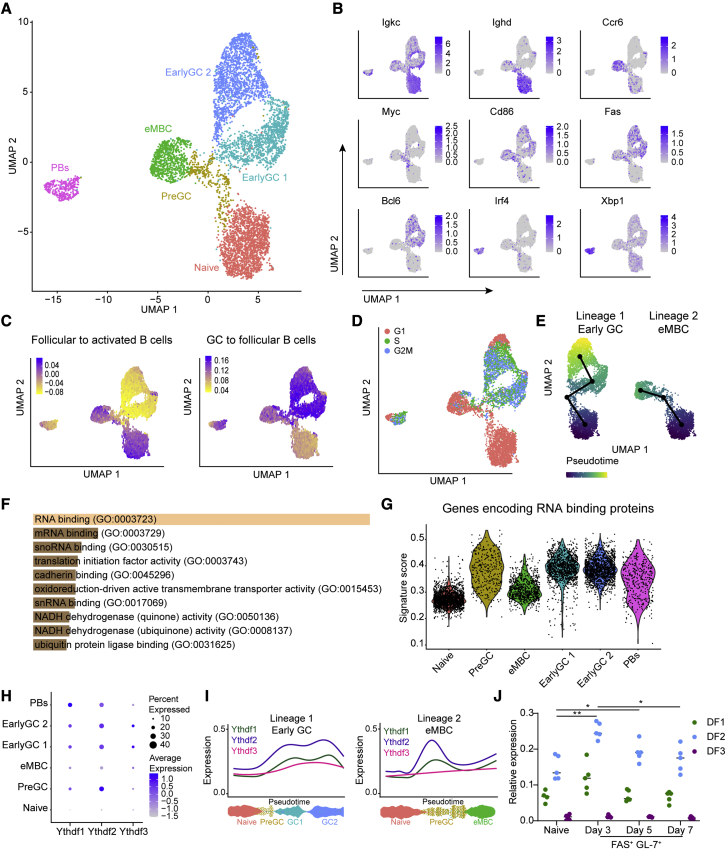
YTHDF2 is upregulated upon B cell activation prior to GC seeding (A) Single-cell RNA-seq of transferred B1-8^hi^ B cells pooled from three mice 5 days after immunization. UMAP projections of scRNA-seq profiles of 6,263 cells. Clusters in the UMAP plots are color coded according to different cell populations. (B) Two-dimensional embedding as in (A) colored to represent the weight of genes associated with distinct cell clusters. (C) UMAP projections of signature scores. Each signature consists of genes that are upregulated (fold change > 2, adjusted p < 0.05) in the indicated cell populations. Gene Expression Omnibus accession numbers GEO: GSE174394 and GSE60927 were used. (D) Cell-cycle phase assignment of single cells based on gene expression. (E) Pseudotime analysis of B cell differentiation trajectories analyzed using Slingshot R package. (F) Molecular function GO terms enriched in genes whose expression is significantly upregulated in pre-GC compared with the naive and eMBC cell clusters. Terms are ordered based on p value ranking. (G) Violin plots of the distribution of RBP gene signature scores in different cell clusters. (H) Dot plot representation of *Ythdf1/2/3* expression arranged by average expression and percent expression per cluster. (I) Expression of *Ythdf1/2/3* along the B cell differentiation trajectories defined in (B), (C), and Figure S1D. (J) qRT-PCR of Ythdf1/2/3 expression in sorted FAS^+^ GL-7^+^ B1-8^hi^ transferred B1-8^hi^ B cells 3–7 days after immunization. Two to three primer pairs per gene were used to measure Ythdf expression. Plot shows average expression relative to a reference gene, Ubc. Data were pooled from five mice from two independent experiments. Lines indicate average mRNA expression. Statistical significance was tested by two-way ANOVA followed by Tukey’s multiple comparisons test. ^∗^p < 0.05, ^∗∗^p < 0.01; ns, not significant.

We wished to investigate mechanisms and gene programs that support B cell fate decisions after the initial antigen encounter but prior to GC formation. To this end, we examined B cell subpopulations during the early stages of the immune response using scRNA-seq. In a polyclonal immune response, the fraction of responding antigen-specific B cells in reactive lymph nodes is very small, and enrichment based on typical activation markers might not capture all of the emerging B cell subsets. To overcome these limitations and capture all of the responding B cells, we adoptively transferred TdTomato^+^ B cells that carry a 4-hydroxy-3-nitrophenyl (NP)-specific B cell receptor (BCR) (B1-8^hi^) (Shih et al., 2002) into wild-type (WT) mice, followed by immunization with NP-KLH. After 5 days and before GC formation, single-cell suspensions from popliteal lymph nodes of three mice were labeled with anti-CD45 antibodies linked to Cell Hashing barcodes, mixed together, and subjected to scRNA-seq as a single pooled sample. Using uniform manifold approximation and projection (UMAP) dimensionality reduction analysis of 6,263 individual cells, we identified six clusters, which were annotated based on their gene expression signatures (Figures 1A, 1B, and S1A). Similar clustering of cells from three individual mice was confirmed, and the data were pooled and examined as a single sample (Figure S1B). One cluster was characterized by high expression levels of *Igkc* and *Ighd* and low levels of *Iglc1*, and since the NP-specific cells express Igλ in B1-8^hi^ transgenic mice (Shih et al., 2002), this cluster was defined as naive B cells that did not respond to the antigen (Figures 1B, 1C, and S1C). Two clusters displayed high levels of *Fas*, *Cd86*, and the GC transcription factor, *Bcl6*, suggesting that these cells were in the process of differentiating into GC B cells (Figures 1B, 1C, and S1D). We also identified a discrete cluster of early PBs expressing high levels of typical PC genes, including *Irf4* and *Xbp1* (Figures 1B and S1D). Another cluster expressed the activation markers *Cd86* and *Fas* as well as high levels of the chemokine receptor *Ccr6*, which is typical of early-forming memory cells (eMBCs) (Figures 1B and S1D) (Schwickert et al., 2011; Taylor et al., 2012; Glaros et al., 2021; Bhattacharya et al., 2007; Laidlaw et al., 2020; Elgueta et al., 2015). Finally, one cluster expressed *Cd86* and *Fas* but lacked markers of GCs and was thus defined as pre-GC cells. Notably, cells in this cluster also expressed high levels of the transcription factor *Myc*, which is essential for early B cell responses and GC seeding, by promoting the transition from G_0_/G_1_ to S phase (Figure 1B) (Calado et al., 2012; Dominguez-Sola et al., 2012). Gene signatures extracted from RNA-seq datasets of purified cell populations were used to validate the cell assignments of each cluster (Figures 1C and S1D). As expected, cell-cycle analysis demonstrated that naive cells and eMBCs are mostly non-cycling cells, whereas some of the pre-GC and most of the early-GC B cells are actively proliferating (Figure 1D). Placement of cells along possible developmental trajectories using pseudo-temporal ordering predicted that the pre-GC cluster was likely to give rise to GC B cells as well as the eMBC cell subset (Figure 1E). In agreement with a recent study showing that differentiation to PBs takes place as early as day 2.5 (Glaros et al., 2021), the PB cluster was well separated from the other clusters at day 5, and PB-differentiating cells were not observed (Figure 1A). Analysis of the expression of several lineage marker genes as a function of pseudotime confirmed that the expression of GC markers increases during cell differentiation to early-GC cells (Figure S1E). Thus, using scRNA-seq, we were able to identify pre-GC and early-GC B cells with the capacity to form mature GCs.

To identify molecular mechanisms that may direct early B cell commitment to GC B cells, we performed an enrichment analysis of genes that are highly expressed in the pre-GC cluster compared with both to the naive and eMBC clusters. Among the 991 genes that met these criteria, 359 genes were annotated as genes encoding RBPs based on molecular function gene ontology (GO) terms (Figure 1F, adjusted p = 3.92e−170, Enrichr). These included genes involved in translation, splicing, RNA transport, and post-transcriptional regulation of gene expression. Gene set enrichment analysis (GSEA) showed elevation of MYC targets as well as genes related to cell cycle and DNA repair (Figure S1F). To further characterize the expression of RBPs during early commitment to GC B cells, we queried all genes annotated as encoding RBPs (GO: 0003723). This analysis confirmed that pre-GC cells, as well as cells of the early-GC cluster, exhibited the highest expression levels of genes encoding RBPs (Figures 1G and S1G). These results suggest that RBPs play a role in early B cell differentiation and GC seeding.

Intriguingly, *Ythdf2*, which encodes an m^6^A binding protein, was one of the genes that met our enrichment criteria (expression significantly increased during the transition from naive to pre-GC cells) (Figure 1H), suggesting that this RNA regulator might facilitate differentiation into GC B cells. Analysis of *Ythdf2* expression as a function of pseudotime revealed that it was upregulated early in the developmental trajectory, starting at pre-GC cells, and was maintained in the early-GC subset (Figures 1H and 1I). The *Ythdf* protein family consists of three paralogs, *Ythdf1*, *Ythdf2*, and *Ythdf3*, that share high levels of sequence homology; however, expression of *Ythdf1* and *Ythdf3* was substantially lower than the expression of *Ythdf2* throughout the B cell lineages (Figure 1I). Consistent with this profile, analysis of sorted B1-8^hi^ B cells using qRT-PCR and multiple primer pairs confirmed that *Ythdf2* is induced early in the immune response and is the dominant paralog expressed in antigen-specific B cells (Figure 1J). These results highlight *Ythdf2* as a potential regulator of the early transition of naive B cells through a pre-GC state to early-differentiated GC cells.

### Antigen-specific B cells located at the lymph node outer follicular regions express YTHDF2

**Figure 2:**
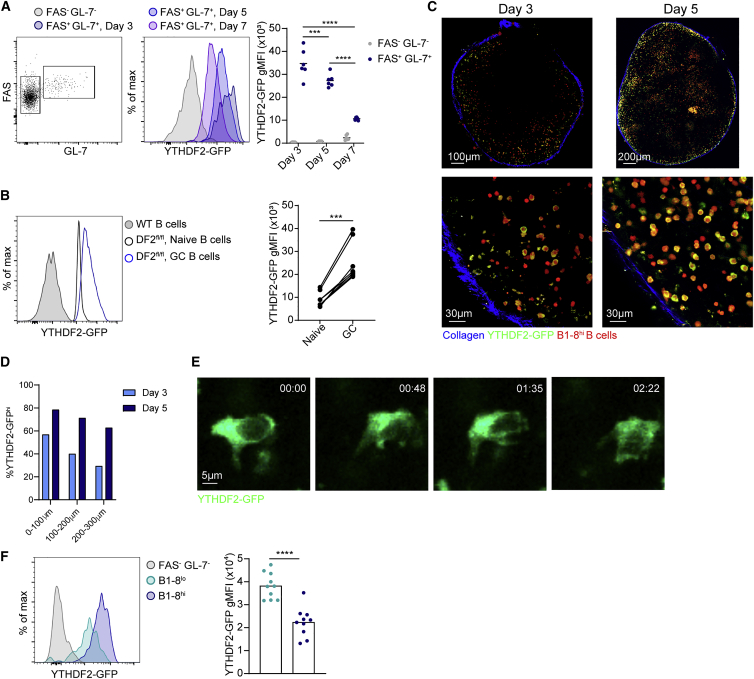
YTHDF2 protein expression is upregulated prior to GC seeding (A) YTHDF2-GFP expression analyzed by flow cytometry in GL-7^−^ FAS^−^ and GL-7^+^ FAS^+^ DF2^fl/fl^ B1-8^hi^ B cells. Data were pooled from six independent lymph nodes from four mice. Lines indicate average protein expression. (B) YTHDF2 expression in naive (FAS^−^ CD38^+^) and GC (FAS^+^ CD38^−^) B cells from WT (gray) and DF2^fl/fl^ mice by flow cytometry. The connected dots represent data from the same mouse. Data were obtained from a total of seven mice from three independent experiments. (C) Images of YTHDF2-GFP expression in TdTomato^+^ DF2^fl/fl^ B1-8^hi^ B cells as in (A) within intact lymph nodes by TPLSM. Images are representative of 3–4 lymph nodes. (D) Quantification of YTHDF2-GFP^hi^ expression fraction in (C). Minimum 360 cells across two lymph nodes were analyzed per genotype. (E) YTHDF2 expression in a moving YTHDF2-GFP^hi^ B1-8^hi^ B cell in a lymph node. (F) YTHDF2-GFP expression was measured in FAS^+^ GL-7^+^ B cells transferred from DF2^fl/+^ B1-8^hi^ and B1-8^lo^ mice, 3 days after NP-KLH immunization. Data were pooled from nine separate lymph nodes from two independent experiments. Statistical significance was tested by two-tailed paired Student’s t test (B) or one-way ANOVA followed by Sidak’s multiple comparisons test (A). ^∗^p < 0.05, ^∗∗^p < 0.01, ^∗∗∗^p < 0.005, ^∗∗∗∗^p < 0.001; ns, not significant.

Next, we determined the expression patterns of YTHDF2 protein in B cells during early B cell activation and GC formation. For this purpose, we used mice that express GFP fused to a floxed *Ythdf2* allele (DF2^fl/fl^) and crossed them to TdTomato^+^ B1-8^hi^ transgenic mice (Shih et al., 2002; Ivanova et al., 2017). To examine the dynamics of YTHDF2-GFP expression at the protein level, we transferred B cells derived from these mice into WT hosts followed by immunization with NP-KLH. Flow-cytometry analysis showed that GL-7^+^ FAS^+^ B cells expressed maximal YTHDF2 protein on day 3 of the response, followed by a gradual decrease on days 5 and 7 after immunization (Figure 2A). Strong upregulation of YTHDF2 in response to vaccination was also observed in a polyclonal immune response that includes B cells expressing immunoglobulins with diverse specificities and affinities (Figures 2B and S2A). We conclude that YTHDF2 is upregulated in antigen-specific B cells early during the immune response and that expression persists during GC seeding.

After the initial cognate antigen encounter, B cells migrate to the border between the T and B cell zones, where they interact with cognate T follicular helper cells (Okada et al., 2005). Subsequently, B cells migrate to the outer follicular regions, where they undergo initial proliferation before returning to the center of the follicle and seed GCs (Green and Cyster, 2012). To examine where B cells express YTHDF2 *in vivo*, we adoptively transferred TdTomato^+^ B1-8^hi^ B cells that express YTHDF2-GFP into host mice. On days 3 and 5 after immunization, popliteal lymph nodes were exposed and imaged using intravital two-photon laser scanning microscopy (TPLSM). On day 3 of the response, ∼50% of the TdTomato^+^ B1-8^hi^ B cells located next to the lymph node capsule expressed high levels of YTHDF2-GFP, and its expression frequency decreased in areas located deeper within the lymph node cortex (Figures 2C and 2D). To make sure that the changes in the GFP signal are not a result of ineffective penetration of the microscope laser into deeper regions of the tissue, we co-transferred TdTomato^+^ B1-8^hi^ B cells that express YTHDF2-GFP and polyclonal CFP^+^ B cells into mice followed by immunization. While the CFP signal did not significantly change, ratiometric measurements of GFP and TdTomato signals revealed a gradual decrease in the YTHDF2-GFP signal intensity when moving away from the lymph node capsule (Figures S2B and S2C). Live imaging of motile B cells revealed that YTHDF2 was found in the cytoplasm of the cells, including both the leading and trailing edge; however, as the trailing edge contains more cytoplasm, most of the protein was located in this area (Figure 2E). To examine whether immunoglobulin affinity affects YTHDF2 expression, we transferred B cells into mice that express low-affinity NP-specific BCRs (B1-8^lo^) and YTHDF2-GFP and compared GFP expression with that of B1-8^hi^ B cells. We found that B1-8^hi^ B cells express higher levels of YTHDF2 compared with their low-affinity counterparts 3 days after immunization with NP-KLH (Figure 2F). Together, these results indicate that YTHDF2 is upregulated in B cells located at the lymph node outer follicular regions and that BCR affinity regulates its expression.

### Germinal center formation depends on YTHDF2

**Figure 3:**
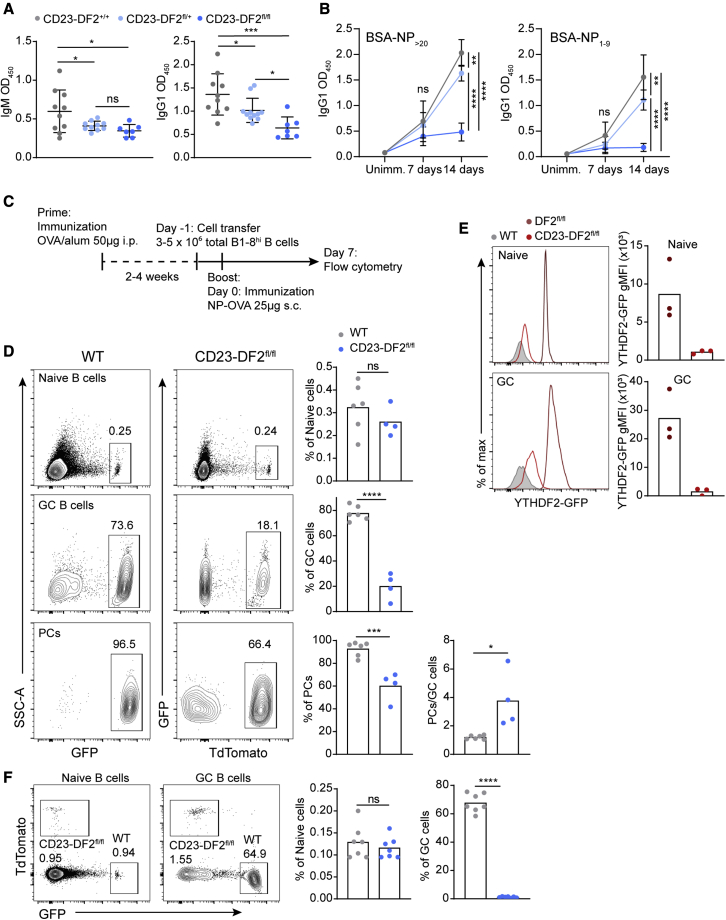
Ythdf2 is required for effective antibody-mediated immune response and GC formation (A) IgM and IgG1 serum titers were determined by ELISA. Serum samples from 7–10 mice were analyzed in a single ELISA assay. Lines represent mean ± SD. O.D., optical density. (B) Mice were immunized with NP-KLH and serum was collected after 7 and 14 days. NP-specific antibodies were measured by ELISA. Data were pooled from a total of 4–11 mice per genotype and time point. Dots represent mean ± SD. (C) Prime-boost immunization protocol. (D) Frequency of naive, GC and PC WT GFP^+^, and CD23-DF2^fl/fl^ TdTomato^+^ B1-8^hi^ B cells 7 days after boosting OVA-primed mice with NP-OVA. Data were pooled from 4–6 mice from two independent experiments. Column heights represent the mean. (E) WT, DF2^fl/fl^, and CD23-DF2^fl/fl^ mice were immunized with NP-KLH, and GFP expression was analyzed after 7 days. Data were pooled from three mice per group. (F) WT GFP^+^ and CD23-DF2^fl/fl^ TdTomato^+^ B1-8^hi^ B cells were mixed at a 1:1 ratio and transferred to OVA-primed WT host mice. Naive and GC cell frequencies were analyzed by flow cytometry 7 days after the boost. Data were pooled from seven mice from three independent experiments. Statistical significance was tested by one-way ANOVA followed by Holm-Sidak multiple comparisons test (A), two-way ANOVA followed by Sidak’s multiple comparisons test (B), or two-tailed unpaired Student’s t test (C and E). ^∗^p < 0.05, ^∗∗^p < 0.01, ^∗∗∗^p < 0.005, ^∗∗∗∗^p < 0.001; ns, not significant.

To determine whether YTHDF2 plays a functional role in the antibody immune response, we crossed Ythdf2^fl/fl^ mice to a B cell-specific CD23-Cre mouse strain (CD23-DF2^fl/+^ and CD23-DF2^fl/fl^). ELISA revealed lower levels of serum IgM and IgG1 in sera of mice that expressed one or two *Ythdf2-flox* copies, demonstrating that this gene is haploinsufficient (Figure 3A). To examine the generation of antigen-specific antibodies, we immunized mice with NP-KLH and analyzed the presence of serum NP-specific antibodies by ELISA 1 and 2 weeks after immunization. CD23-DF2^fl/fl^ mice generated low levels of antibodies on day 7 of the response during GC formation compared with their WT counterparts; however, this difference was not statistically significant (Figure 3B). After GC formation on day 14 of the response, very low levels of antigen-specific antibodies were detected in the CD23-DF2^fl/fl^, whereas CD23-DF2^fl/+^ produced IgG1 that bound both total and low-density NPs (NP_20_-BSA, and NP_1-9_-BSA, respectively) (Figure 3B). Collectively, these experiments show that an effective antibody-mediated immune response depends on YTHDF2.

Since GCs are the primary source of antibody-forming cells, we next sought to determine whether GC formation depends on YTHDF2. To this end we used a prime-boost protocol, which allows the analysis of the immune response without the effects of adjuvants. WT mice were first primed by intraperitoneal OVA injection followed by adoptive cell transfer of either CD23-DF2^fl/fl^ or WT B1-8^hi^ naive B cells and boosted with NP-OVA (Figure 3C) (Schwickert et al., 2011). Flow-cytometry analysis of popliteal lymph node-derived cells 7 days after the boost showed that the fraction of adoptively transferred CD23-DF2^fl/fl^ B1-8^hi^ B cells that differentiated into GC cells was 3.9-fold lower than that of control cells (Figures 3D and S2A). The reduction in the frequency of PCs was less pronounced (1.5-fold lower) and the PC/GC ratio of Ythdf2-deficient B cells was higher compared with control cells (Figure 3D). The few CD23-DF2^fl/fl^ B1-8^hi^ GC B cells did not show expression of YTHDF2-GFP by flow-cytometry analysis (Figure 3E). Similarly, a small GC cell population was detected in a polyclonal immune response by immunization of CD23-DF2^fl/fl^ mice (Figure S2D). During the early stages of the immune response, B cells compete for GC seeding, and occasionally the functions of a particular molecule can be revealed only under competitive pressure (Zaretsky et al., 2017; Schwickert et al., 2011). To further examine the significance of YTHDF2 in GC seeding, we transferred a mix of WT and Ythdf2-deficient B1-8^hi^ B cells into WT hosts before administration of the NP-OVA boost. Flow-cytometry analysis 7 days later revealed that under competitive conditions, Ythdf2-deficient B1-8^hi^ B cells were completely blocked in their ability to differentiate into GC cells (Figure 3F). We conclude that the reduction in serum antibody levels is primarily a result of defects in GC formation by Ythdf2-deficient B cells. Collectively, these findings demonstrate that YTHDF2 is essential for generation of intact antibody-mediated immune responses, primarily by facilitating GC formation.

### YTHDF2 is required for germinal center seeding but not for early B cell activation

**Figure 4:**
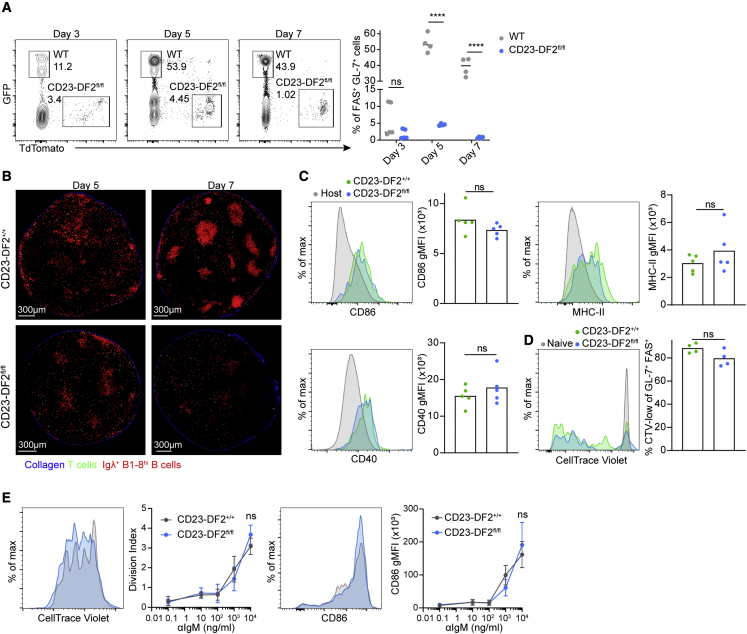
YTHDF2 is required for early stages of the B cell immune responses but not for initial B cell activation (A) WT GFP^+^ and TdTomato^+^ CD23-DF2^fl/fl^ B1-8^hi^ B cells were transferred to WT host mice at a 1:1 ratio. The frequencies of FAS^+^ GL-7^+^ cells were analyzed by flow cytometry 3, 5, and 7 days after NP-KLH immunization. (B) CD23-DF2^+/+^ and CD23-DF2^fl/fl^ B1-8^hi^ TdTomato^+^ Igλ^+^ B cells and GFP^+^ CD4^+^ T cells were transferred separately to WT host mice. Lymph nodes were imaged by TPLSM 5 and 7 days after NP-KLH immunization. Images are representative of 3–4 independently analyzed lymph nodes. (C) CD86, CD40, and MHC-II expression on transferred Igλ^+^ B1-8^hi^ B cells were analyzed 16 h after immunization by flow cytometry. Data were pooled from five mice from two independent experiments. (D) B1-8^hi^ B cells were stained with CellTrace Violet prior to cell transfer and its dilution was measured 3 days after immunization by flow cytometry. (E) Splenic cells derived from CD23-DF2^+/+^ and CD23-DF2^fl/fl^ mice were stained with CTV and stimulated with 0.1–10^4^ ng/mL αIgM for 3 days *in vitro*. Division index was calculated based on CTV dilution. CD86 expression was examined in similar manner. Plots are averaged from three independent experiments each performed with three replicates per condition. Dots represent mean ± SD. Statistical significance was tested by one-way ANOVA followed by Holm-Sidak multiple comparisons test (A), two-way ANOVA (E), or two-tailed unpaired Student’s t test (C and D). ^∗∗^p < 0.01, ^∗∗∗∗^p < 0.001; ns, not significant.

To evaluate whether YTHDF2 is required at the early stages of the B cell immune response, we adoptively transferred WT and Ythdf2-deficient B1-8^hi^ B cells together into host mice and examined the presence of FAS^+^ GL-7^+^ B cells by flow cytometry 3–7 days after immunization with NP-KLH. This analysis revealed that the frequency of Ythdf2-deficient B cells was significantly reduced at day 5 of the immune response before GC formation (Figure 4A). Although the response at day 3 was very weak, the mice with the higher fraction of responding B1-8^hi^ B cells had fewer activated CD23-DF2^fl/fl^ cells than activated control cells, suggesting a defect at day 3 as well (Figure 4A). B cell immune responses involve changes in the location of the responding cells within the lymph node, which promotes proper differentiation (Green and Cyster, 2012). To examine how Ythdf2-deficiency affects B cell positioning, we scanned intact lymph nodes using TPLSM. On day 5 after immunization, CD23-DF2^+/+^ B1-8^hi^ B cells were observed at the lymph node cortex, and on day 7, clear GC structures were detected (Figure 4B; Videos S1 and S2). Cell clusters were detected in mice that received CD23-DF2^fl/fl^ B1-8^hi^ B cells on day 5 of the response; however, mature GCs were not formed 2 days later, suggesting that YTHDF2 is required for GC seeding (Figure 4B; Videos S3 and S4).

Video S1. CD23-DF2+/+ B1-8hi TdTomato+ Igλ+ B cells and GFP+ CD4+ T cells in WT host mice 5 days after NP-KLH immunization, related to Figure 4 Scale bar, 150 μm.

Video S2. CD23-DF2fl/fl B1-8hi TdTomato+ Igλ+ B cells and GFP+ CD4+ T cells in WT host mice 5 days after NP-KLH immunization, related to Figure 4 Scale bar, 150 μm.

Video S3. CD23-DF2+/+ B1-8hi TdTomato+ Igλ+ B cells and GFP+ CD4+ T cells in WT host mice 7 days after NP-KLH immunization, related to Figure 4 Scale bar, 150 μm.

Video S4. CD23-DF2fl/fl B1-8hi TdTomato+ Igλ+ B cells and GFP+ CD4+ T cells in WT host mice 7 days after NP-KLH immunization, related to Figure 4 Scale bar, 150 μm.

To specifically determine whether YTHDF2 plays a role in the course of GC seeding rather than during initial activation by cognate antigen, we examined whether Ythdf2-deficient B cells are able to respond to an antigen *in vivo*. Upregulation of typical activation markers such as CD86, MHC-II, and CD40 was detected on Ythdf2-deficient B cells 16 h after immunization (Figure 4C). Furthermore, tracking the dilution of CellTrace Violet in responding B cells revealed that there were no early B1-8^hi^ B cell proliferation defects (Figure 4D). In addition, stimulation of B cells *in vitro* by increasing doses of anti-IgM did not reveal any notable defects in proliferation or CD86 expression in Ythdf2-deficient B cells (Figure 4E). Thus, B cell activation and proliferation triggered by BCR engagement do not depend on YTHDF2.

### YTHDF2 suppresses the plasmablast genetic program during GC seeding

**Figure 5:**
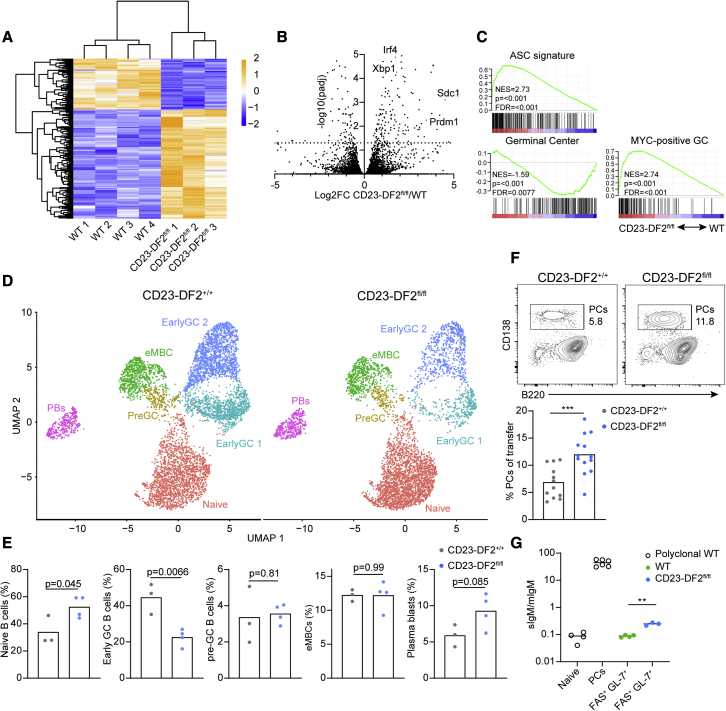
The plasmablast genetic program is repressed by YTHDF2 (A) Heatmap representation of clustering analysis of differentially expressed genes in transferred WT and CD23-DF2^fl/fl^ FAS^+^ GL-7^+^ B1-8^hi^ B cells sorted 5 days after immunization. (B) Volcano plot of statistical significance against fold change, demonstrating the most significantly differentially expressed genes. PB-associated genes are marked in blue. Log2FC, Log_2_ fold change. (C) GSEA of differentially expressed genes. Signatures were derived from public RNA-seq datasets (MYC-positive GC, GEO: GSE39443; GC, GEO: GSE110669; ASC, GEO: GSE60927). (D) UMAP projections of scRNA-seq profiles of 6263 CD23-DF2^+/+^ and 4964 CD23-DF2^fl/fl^ B1-8^hi^ B cells 5 days after immunization. Data were pooled from 3–4 host mice per genotype. Clusters in the UMAP plots are color coded according to different cell populations. Annotation of cell populations was based on the same markers as in Figures 1B, 1C, and S1D. (E) Quantification of scRNA-seq profiles per cluster based on Cell Hashing replicates. (F) Analysis of PC formation potential of transferred B1-8^hi^ B cells 5 days after NP-KLH immunization. Data were pooled from 12–14 mice from five independent experiments. (G) qRT-PCR analysis of mIgM and sIgM mRNA transcripts derived from transferred WT and CD23-DF2^fl/fl^ FAS^+^ GL-7^+^ B1-8^hi^ B cells as in (A), as well as from polyclonal WT naive B cells and PCs. Data were pooled from a total of 3–6 mice. Lines indicate the mean. Statistical significance was tested by two-tailed unpaired Student’s t test (E and F) or one-way ANOVA followed by Holm-Sidak multiple comparisons test (G). ^∗∗∗^p < 0.005.

The discrepancy between the *in vivo* experiments that show a substantial dependence on YTHDF2 for GC seeding and the lack of perturbations in early B cell activation and proliferation in the absence of YTHDF2 suggests that this m^6^A reader plays a role in B cell differentiation and fate decision rather than in cell-cycle progression and clonal expansion. To further evaluate the stages at which YTHDF2 is essential for B cell development, we examined genetic programs that support B cell fate commitment. To this end, we sorted WT and Ythdf2-deficient B1-8^hi^ B cells that responded to the antigen from recipient hosts 5 days after immunization and subjected them to bulk RNA-seq. Analysis of differential gene expression revealed that 159 genes were significantly upregulated while 69 genes were significantly downregulated in Ythdf2-deficient B cells (log_2_ fold change ≥ ±0.58, adjusted p ≤ 0.05) (Figures 5A and 5B). The higher proportion of upregulated genes in Ythdf2-deficient B cells is consistent with the RNA-degradation-enhancing function of YTHDF2 (Du et al., 2016). Strikingly, genes typically expressed in PB and PCs, such as *Irf4*, *Xbp1*, *Sdc1*, and *Prdm1*, were highly expressed in Ythdf2-deficient GL-7^+^ FAS^+^ B cells (Figure 5B). These changes were reflected in the increased expression of the ASC gene signature as revealed by GSEA (Figure 5C). Furthermore, and consistent with the functional results, the GC gene signature was significantly downregulated in Ythdf2-deficient B cells. Although MYC is important for GC seeding, the MYC gene signature was increased in Ythdf2-deficient B cells, which do not effectively seed GCs (Figure 5C). This effect is most likely due to the role of YTHDF2 in the suppression of *Myc* expression through direct interaction with *Myc* mRNA (Grenov et al., 2021; Dixit et al., 2021). In line with these results, we observed a trend in increased Myc signature and ASC signature in Ythdf2-deficient GC B cells using the AID-Cre mouse model (Figure S2E) (Grenov et al., 2021). Taking these data together, we conclude that YTHDF2 suppresses the PB genetic program at the early stages of the B cell immune response.

To further examine cell fates of early-responding B cells in the absence of YTHDF2, we performed scRNA-seq analysis. For this purpose, Ythdf2-deficient and control B1-8^hi^ B cells were sorted from four and three mice, respectively, at day 5 after immunization and analyzed by scRNA-seq. Since a similar number of cells were sequenced from each mouse strain, the scRNA-seq analysis does not represent the actual number of responding cells in the mice but rather the frequencies of each cell type within the adoptively transferred B cells. A combined UMAP analysis of a total of 11,227 cells revealed clustering similar to that described in Figure 1 (Figure 5D). However, whereas Ythdf2-deficient B cells differentiated into pre-GC and eMBC subsets, they formed significantly fewer early-GC B cells (Figure 5E). In contrast, the frequency of Ythdf2-deficient PBs was increased but did not reach statistical significance due to the low number of scRNA-seq replicates. We conclude that YTHDF2 facilitates GC seeding, whereas differentiation into early PBs does not require YTHDF2 expression.

To complement the single-cell data, we examined the potential of adoptively transferred Ythdf2-deficient B1-8^hi^ B cells to form PBs on day 5 of the response using flow cytometry. In this analysis, we gated on total adoptively transferred B1-8^hi^ B cells rather than comparing cell frequencies within each cell subset. Consistent with the transcriptomic analyses, we found that the responding Ythdf2-deficient B cells form relatively more PCs than control B cells (Figure 5F). Thus, YTHDF2 suppresses the PB genetic program in early-responding B cells and increases their probability to differentiate into GC B cells.

Next, we examined whether early FAS^+^ GL-7^+^ Ythdf2-deficient B cells possess PB-related functions in addition to the increased ASC gene expression. An important hallmark of B cell differentiation to ASCs is the alternative splicing switch to form secreted immunoglobulins rather than membrane-bound B cell receptors. To examine whether YTHDF2 plays a functional role in early-responding antigen-specific B cells as a result of the suppression ASC genetic program, we measured the relative expression of secreted and membrane-bound immunoglobulin transcripts (sIgM and mIgM, respectively) in control and Ythdf2-deficient B cells. To this end, we sorted FAS^+^ GL-7^+^ B cells from immunized mice and performed qRT-PCR using primers specific for each immunoglobulin isoform. To ensure detection of isoform-specific transcripts, we used sorted polyclonal naive B cells as a negative control and PCs as a positive control. As expected, naive B cells showed very low if any sIgM transcripts, whereas PCs showed a very high ratio of sIgM to mIgM transcripts (Figure 5G). Analysis of WT B1-8^hi^ B cells isolated 5 days after immunization revealed a ratio of sIgM to mIgM similar to that of naive cells, demonstrating that these cells do not produce antibodies. In contrast, Ythdf2-deficient B cells showed a 3-fold increase in the sIgM/mIgM ratio (Figure 5G), suggesting that they produce secreted IgM transcripts although they did not differentiate to PBs. These results provide additional evidence for the role of YTHDF2 in the suppression of the PB genetic program at the pre-GC and early-GC stages.

### YTHDF2 directly interacts with methylated mRNAs of plasmablast-regulating genes

**Figure 6:**
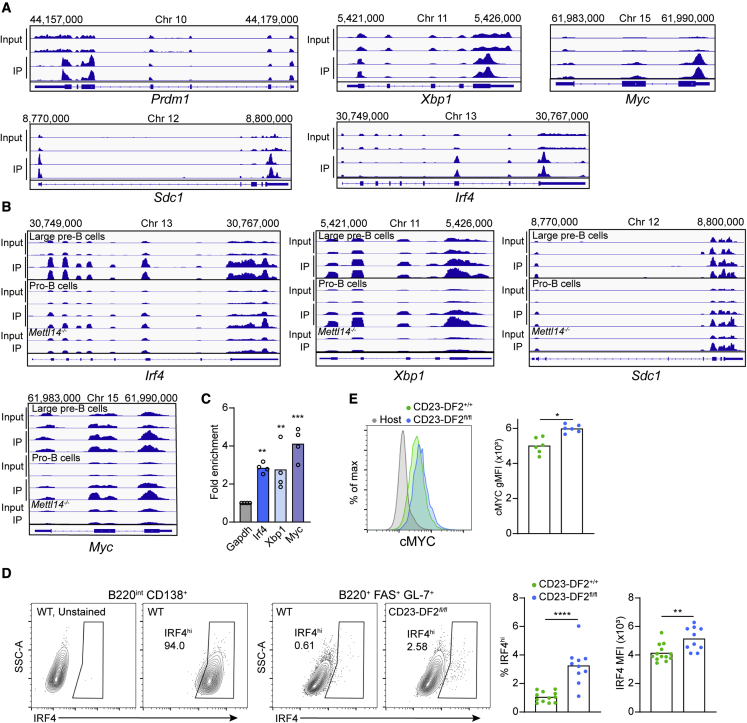
Plasma blast-regulating gene transcripts are direct targets of YTHDF2 (A) m^6^A peak profiles of representative PB-associated genes analyzed by m^6^A-IP followed by high-throughput sequencing. (B) Representation of YTHDF2-IP profiles from public dataset GEO: GSE174394 of *Irf4*, *Xbp1*, and *Myc*. (C) RIP of HA-YTHDF2-GFP performed using LPS-stimulated B cells derived from DF2^fl/fl^ mice (HA-YTHDF2-GFP^+/+^, Cre^−^). Fold enrichment of RNA in pull-down fraction over input fraction normalized to unmethylated *Gapdh* RNA and an isotype control. Pooled data from four replicates from two independent experiments. (D and E) CD23-DF2^+/+^ and CD23-DF2^fl/fl^ B1-8^hi^ B cells were isolated and transferred to WT host mice. Five days after immunization with NP-KLH, GL-7^+^ FAS^+^ B1-8^hi^ B cells were analyzed for IRF4 (D) and MYC (E) protein expression by intracellular staining and flow cytometry. Pooled data from 6–12 mice from 2–4 independent experiments. Statistical significance was tested using a two-tailed unpaired t test (D) or one-way ANOVA followed by Holm-Sidak multiple comparisons test (G). ^∗^p < 0.05, ^∗∗^p < 0.01, ^∗∗∗^p < 0.005, ^∗∗∗∗^p < 0.001.

The YTHDF protein family promotes the degradation of specific mRNAs by binding m^6^A-modified transcripts and interacting with RNA deadenylation complexes (Zaccara et al., 2019). Since our results indicated a role of YTHDF2 in repressing PB genetic programs, we sought to determine whether this protein can directly target PB-promoting genes. To this end, we first generated cells *in vitro* that express PB genes by stimulating isolated B cells with lipopolysaccharide (LPS) for 3 days. We then performed RNA immunoprecipitation (IP) using an anti-m^6^A antibody and used high-throughput sequencing as a readout (Dominissini et al., 2012; Grenov et al., 2021). A comparison of the IP with input fractions revealed that several PC differentiation genes that were upregulated in Ythdf2-deficient B cells, including *Prdm1*, *Sdc1*, *Xbp1*, and *Irf4*, showed one or more m^6^A peaks (Figure 6A). Furthermore, through the analysis of a published dataset of YTHDF2 IP performed in developing B cells (Zheng et al., 2020) we found that both *Irf4* and *Xbp1* transcripts are directly bound by YTHDF2 (Figure 6B). According to this analysis, *Sdc1* was also bound by YTHDF2; however, this peak persisted in cells lacking the mRNA methylation machinery, indicating non-specific binding. Similar results were obtained by RNA immunoprecipitation of HA-YTHDF2-GFP from LPS-stimulated transgenic B cells followed by qRT-PCR (Figure 6C). These findings suggest that YTHDF2 has the potential to directly modulate PC differentiation genes at the post-transcriptional level.

Among the PB-related genes that were upregulated in the early-responding B cells, *Irf4* is considered the most upstream B cell fate regulator (Nutt et al., 2011). Analysis of IRF4 protein expression in CD23-DF2^+/+^ and CD23-DF^fl/fl^ cells 5 days after immunization demonstrated that the frequency of activated CD138-negative B cells that show high levels of IRF4 was 2.5-fold higher in CD23-DF^fl/fl^ cells compared with the control, and increased levels of protein per cell were detected by of mean fluorescence intensity measurement (Figure 6D). A small increase in MYC protein levels was also detected in activated and YTHDF2-deficient B cells (Figure 6E) (Grenov et al., 2021). These findings indicate that YTHDF2 has the potential to directly regulate PC formation through the modulation of the mRNA and protein expression levels of IRF4.

### Germinal center and plasmablast formation do not depend on YTHDF1 or YTHDF3

**Figure 7:**
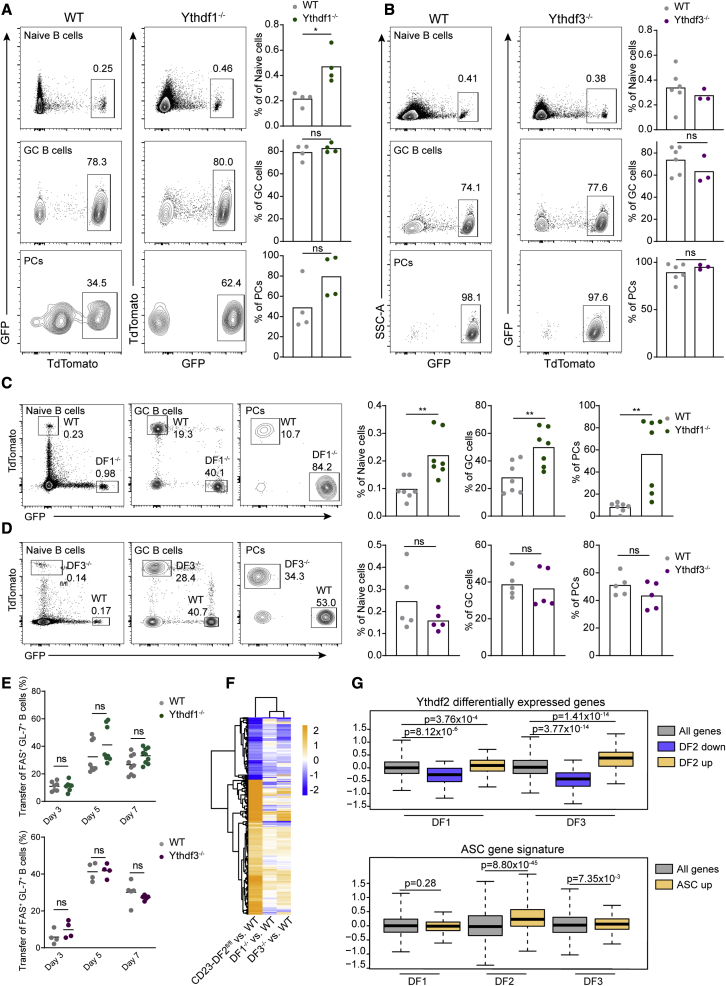
Germinal center formation does not depend on YTHDF1 and YTHDF3 (A–D) Ythdf1^−/−^ GFP, Ythdf3^−/−^ TdTomato^+^ B1-8^hi^ B cells, WT GFP, or TdTomato^+^ B1-8^hi^ B cells were transferred to OVA-primed WT host mice, separately (A and B) or mixed at a 1:1 ratio (C and D). Lymph node cells were analyzed by flow cytometry 7 days after the boost. Pooled data from 3–6 mice from two independent experiments. Column heights represent the mean. (E) Frequency of transferred FAS^+^ GL-7^+^ WT, GFP^+^ Ythdf1^−/−^ or TdTomato^+^ Ythdf3^−/−^ B1-8^hi^ B cells in immunized mice. Pooled data from 4–9 mice from 2–3 independent experiments. (F) RNA-seq analysis of WT, Ythdf1^−/−^, and Ythdf3^−/−^ B1-8^hi^ B cells 5 days after immunization. Heatmap showing log_2_-transformed fold changes of genes in Ythdf1-, 2- or 3-deficient cells compared with WT cells. All genes that were significantly differentially expressed in Ythdf2-deficient B cells compared with WT cells are shown. (G) Box plots indicating the median, quartiles, and 5th and 95th percentiles of changes in expression levels of Ythdf1^−/−^, Ythdf3^−/−^, and CD23-Ythdf2 compared with WT B1-8^hi^ B cells, of genes which were either down- or upregulated in Ythdf2-deficient cells or of genes which were upregulated in ASC (GEO: GSE60927). Statistical significance was tested using a two-tailed unpaired t test (A–D), one-way ANOVA followed by Holm-Sidak multiple comparisons test (E), or a two-sided Wilcoxon rank-sum test (G). ^∗^p < 0.05, ^∗∗^p < 0.01; ns, not significant.

The YTHDF proteins are structurally very similar (Zaccara and Jaffrey, 2020), and several studies have suggested that all three paralogs promote RNA degradation and can compensate for the loss of any paralog (Lasman et al., 2020; Zaccara and Jaffrey, 2020). Since our results showed that one allele of *Ythdf2* is insufficient for an intact antibody-mediated immune response, we tested whether YTHDF1 and YTHDF3 also contribute to GC formation. To examine B cell-specific immune responses, we bred Ythdf1^−/−^ and Ythdf3^−/−^ mice to the B1-8^hi^ mouse model and used the prime-boost protocol to study the response to NP-OVA. As opposed to Ythdf2-deficient B cells, adoptively transferred Ythdf1- and Ythdf3-deficient B1-8^hi^ B cells efficiently generated both GC cells and PCs (Figures 7A and 7B). Ythdf1-deficient B cells more effectively formed PCs, most likely due to an increase in naive cell frequency in lymph nodes (Figure 7A). Notably, Ythdf1-deficient cells expressed higher levels of CD62L, a molecule that plays a key role in entry into lymph nodes. Other functions in Ythdf1-deficient B cells such as CD86 expression and proliferation were intact (Figures S2F and S2G). Higher frequencies of Ythdf1-deficient PCs and GC cells were detected under competitive conditions, whereas Ythdf3-deficient B cells showed no changes in the frequency of these cell subsets (Figures 7C and 7D). These results suggest that in contrast to YTHDF2, the YTHDF1/3 paralogs do not facilitate GC B cell formation through contribution to the total YTHDF protein pool in B cells. Flow-cytometric analysis of B1-8^hi^ B cells on days 3, 5, and 7 after immunization showed that neither YTHDF1 nor YTHDF3 was required for the formation of FAS^+^ GL-7^+^ B1-8^hi^ B cells (Figure 7E). Nevertheless, RNA-seq experiments of FAS^+^ GL-7^+^ B1-8^hi^ B cells derived from immunized mice revealed that genes which were significantly differentially expressed in Ythdf2-deficient B cells were similarly altered in the absence of Ythdf1 and Ythdf3, although to a lesser extent (Figures 7F and 7G). Although we did not detect increased levels of IRF4 in gene expression datasets of Ythdf1- and Ythdf3-deficient B cells, an increase in ASC signature was observed in Ythdf3-deficient B cells (Figure 7G). Collectively, based on our findings and previous studies that examined YTHDF paralogs in other cell types (Zaccara and Jaffrey, 2020; Lasman et al., 2020), we conclude that YTHDF2 plays a dominant role in B cells, owing to its high expression in these cells.

## Discussion

Differentiation of B cells and fate commitment during the initial stages of the antibody immune response is directed by multiple transcription factors that promote specific cell fates. Early-responding B cells that compete for T cell help at the pre-GC stage express activation markers including FAS and GL-7 (Schwickert et al., 2011; Zaretsky et al., 2017; Yeh et al., 2018). In addition, CCR6 was believed to also define the pre-GC cell subset (Schwickert et al., 2011). Using single-cell analysis, Glaros et al. (2021) described a tri-potent cell subset that gave rise to activated precursors; however, these cells did not show high expression levels of CCR6. In contrast, the CCR6-positive B cells detected early during the response were defined as early memory cells. Our scRNA-seq analysis of the early stages of the B cell response is largely consistent with these findings, and the minor observed differences are most likely due to differences in immunization protocols. Furthermore, by analyzing gene expression at specific B cell differentiation stages, we found that many RBPs, including YTHDF2, are upregulated in early-responding antigen-specific B cells during the pre-GC stage. This observation indicates that post-transcriptional events play a key role in early B cell differentiation (Díaz-Muñoz and Turner, 2018). The finding that Ythdf2-deficient B cells at the pre-GC and early-GC stages showed increased expression of typical PB genes suggests that YTHDF2 reinforces the GC fate by mitigating the PB genetic program at the mRNA level during GC seeding. Thus, in addition to the well-described transcription factor-driven mechanism, we describe a post-transcriptional regulatory pathway that directs B cell differentiation and fate commitment.

For a naive or activated B cell to differentiate into a PB or a GC cell it has to rewire its genetic program, a process mediated by chromatin remodelers and transcription activators and repressors (Nutt et al., 2015; Cook et al., 2020; Schmiedel et al., 2021). Although YTHDF2 has the potential to promote PB formation through multiple targets, our findings indicate that one of the functions of YTHDF2 is to suppress the expression of *Irf4* and other PB-related genes such as *Prdm1* and *Xbp1* and thereby support the stabilization of the GC genetic program. Thus, our observations suggest that YTHDF2 contributes to the “kinetic model,” which proposes that the expression level of IRF4 determines B cell fate early during the response (Ochiai et al., 2013). *Myc* mRNA, a key gene in the B cell immune response, is a direct target of YTHDF2, which facilitates its mRNA degradation and suppresses downstream activation of MYC-responsive genes (Su et al., 2018; Grenov et al., 2021). Since Blimp1 suppresses Myc expression, excess Myc RNA in YTHDF2-deficient B cells might interfere with cell fate trajectories of the responding B cells as well (Karube and Campo, 2015). In our previous analysis of gene expression in the GC, we found that YTHDF2 deletion was associated with a reduction in expression of the oxidative phosphorylation genetic program (Grenov et al., 2021). Since YTHDF2 suppresses gene expression, this downregulation in mRNA levels in YTHDF2-deficient B cells suggests that the effect occurs in an indirect manner. In the current study, we examined pre-GC B cells and found an increase in the PC genetic program in YTHDF2-deficient cells, and accordingly, a reanalysis of the dataset from the previous study also shows similar trends. It is important to note that GC and pre-GC B cells have different metabolic requirements, and GC B cells cannot efficiently support glycolysis (Weisel et al., 2020; Biram et al., 2022). Thus, we suggest that the PC program is directly regulated by YTHDF2, whereas the metabolic changes are specific to GC B cells and represent downstream effects.

In a recent study, it was suggested that YTHDF2 supports PC accumulation *in vitro* (Turner et al., 2022). In mice,the authors show that YTHDF2 is required for an intact GC response on day 21 after immunization in chimeric mice, whereas GCs on day 7 seem to be intact. Although the different mouse models in the two studies might have affected the immune response kinetics, both studies show a reduction in GC size. To strengthen our conclusions, in the present study gene profiles that support PC and GC formation and cell identity were extensively examined, and the conclusions about the suppressive role of YTHDF2 in PC formation are supported by both these analyses and flow-cytometric data. Furthermore, in both of the studies *Irf4* and *Prdm1* transcripts were found to be methylated, and we show that PC-promoting transcripts are bound by YTHDF2, which strongly suggests that these genes are targets for YTHDF2-mediated mRNA degradation. Lastly, we demonstrate that YTHDF2 is required for GC formation both in a polyclonal and in an antigen-specific response using transgenic B cells. B1-8^hi^ B cells were found to express high levels of YTHDF2 compared with their low-affinity counterparts, suggesting that YTHDF2 plays a more significant role in suppression of the PC program in B cells that express high-affinity BCRs and thereby direct their differentiation to GC B cells.

Similar to B cells, the terminal maturation of multiple immune cell types, including natural killer, Tfh, and dendritic cells, depend on mRNA methylation (Yao et al., 2021; Ma et al., 2021; Bechara et al., 2021; Han et al., 2019). Hematopoietic stem cell (HSC) differentiation is highly dependent on m^6^A machineries (Mapperley et al., 2021; Lee et al., 2019; Zaccara et al., 2019) and scRNA-seq of HSCs deficient in m^6^A machineries revealed the development of an intermediate differentiation cell state that cannot properly support hematopoiesis (Cheng et al., 2019). Collectively, together with our results regarding B cell fate decisions, these findings suggest that the m^6^A machinery plays a key role in directing lineage commitments of multiple cell types.

Although it was initially thought that the different YTHDF paralogs have distinct biological activities, recent data suggested that they have fully overlapping functions (Zaccara and Jaffrey, 2020; Lasman et al., 2020). Our transcriptomic analysis of Ythdf1/2/3-deficient B cells shows that the three paralogs share similar patterns of gene regulation, although changes in gene expression are more pronounced in Ythdf2-deficient cells. Based on our findings and previous studies that examine YTHDF paralogs in other cell types, we conclude that YTHDF2 plays a dominant role in B cells because it is highly expressed in these cells.

In summary, in addition to the well-studied transcription factor-mediated control of early B cell fate decisions, our study exposes a post-transcriptional mechanism that directs B cell fate commitment. Our findings suggest that YTHDF2 inhibitors may be beneficial in the treatment of lymphomas that specifically originate from the GC reaction (Victora et al., 2012).

### Limitations of the study

We find that YTHDF2 suppresses the expression of genes that support PC formation; however, regulation of additional genes supporting additional pathways and fate decisions may also play a role.

## STAR★Methods

### Key resources table

**Table:** 

REAGENT or RESOURCE	SOURCE	IDENTIFIER
**Antibodies**		

CD45R (B220) Monoclonal Antibody (RA3-6B2), APC-eFluor 780	eBioscience	Cat# 47-0452-82, RRID: AB_1518810
CD45R (B220) Monoclonal Antibody (RA3-6B2), eFluor 450	eBioscience	Cat# 48-0452-82, RRID: AB_1548761
APC anti-mouse/human CD45R/B220 Antibody (RA3-6B2)	BioLegend	Cat# 103212, RRID: AB_312997
Brilliant Violet 605™ anti-mouse CD138 (Syndecan-1) Antibody (281–2)	BioLegend	Cat# 142516, RRID: AB_2562337
CD38 Monoclonal Antibody (90), Alexa Fluor 700	eBioscience	Cat# 56-0381-82, RRID: AB_657740
F4/80 Monoclonal Antibody (BM8), APC-eFluor 780	eBioscience	Cat# 47-4801-82, RRID: AB_2735036
PE-Cy™7 Hamster Anti-Mouse CD95 (Jo2)	BD	Cat# 557653, RRID: AB_396768
Alexa Fluor® 647 anti-mouse/human GL7 Antigen Antibody (GL7)	BioLegend	Cat# 144606, RRID: AB_2562185
FITC anti-mouse/human GL7 Antigen Antibody (GL7)	BioLegend	Cat# 144604, RRID: AB_2561697
PerCP/Cyanine5.5 anti-mouse/human GL7 Antigen (GL7)	BioLegend	Cat# 144610, RRID: AB_2562979
Ly-6G/Ly-6C Monoclonal Antibody (RB6-8C5), APC-eFluor 780	eBioscience	Cat# 47-5931-82, RRID: AB_1518804
APC anti-mouse Ig light chain λ Antibody (RML-42)	BioLegend	Cat# 407306, RRID: AB_961363
PE anti-mouse CD86 Antibody (GL-1)	BioLegend	Cat# 105008, RRID: AB_313151
PE/Cyanine7 anti-mouse CD86 Antibody (GL-1)	BioLegend	Cat# 105014, RRID: AB_439783
FITC anti-mouse CD86 Antibody (GL-1)	BioLegend	Cat# 105006, RRID: AB_313149
BV421 Rat Anti-Mouse CD40 (3/23)	BD	Cat# 562846, RRID: AB_2734767
Brilliant Violet 510™ anti-mouse I-A/I-E Antibody (M5/114.15.2)	BioLegend	Cat# 107636, RRID: AB_2734168
Alexa Fluor® 647 anti-IRF4 Antibody (IRF4.3E4)	BioLegend	Cat# 646408, RRID: AB_2564048
CD4 Monoclonal Antibody (GK1.5), APC-eFluor 780	eBioscience	Cat# 47-0041-82, RRID: AB_11218896
CD8a Monoclonal Antibody (53–6.7), APC-eFluor 780	eBioscience	Cat# 47-0081-82, RRID: AB_1272185
Anti-HA.11 Epitope Tag Antibody (16B12)	eBioscience	Cat# 901515, RRID: AB_2565334
Goat Anti-Mouse IgM mu chain (HRP)	Abcam	Cat# ab97230
Goat Anti-Mouse IgG1 (HRP)	Abcam	Cat# ab97240
c-Myc (D84C12) Rabbit mAb	Cell Signaling	Cat# 5605

**Chemicals**, **peptides**, **and recombinant proteins**		

Fast SYBR™ Green Master Mix	Applied Biosystems	Cat# 4385614
NP-OVAL (Ovalbumin)	Biosearch Technologies	Cat# N-5051-100
NP-KLH (Keyhole Limpet Hemocyanin)	Biosearch Technologies	Cat# N-5060-25
Imject Alum Adjuvant	Thermo Fisher Scientific	Cat# 77161
Albumin from chicken egg white (OVA)	Sigma-Aldrich	Cat# A5503
qScript cDNA Synthesis Kit	QuantaBio	Cat# 95047
CellTrace™ Violet Cell Proliferation Kit, for flow cytometry	Molecular Probes	Cat# C34557

**Critical commercial assays**		

Chromium Single Cell 30 reagent kits v1	10xGenomics	Cat# PN-1000268
Fixation/Permeablization Kit	BD	Cat# 554714

**Deposited data**		

B1-8hi Ythdf1/Ythdf2/Ythdf3-deficient B cells	This paper	GEO: GSE189819
Follicular B cells, activated B cells, MBCs, and GC B cells	Viant et al., 2021	GEO: GSE174394
Antibody secreting cell signature	Shi et al., 2015	GEO: GSE60927
MYC positive B cells signature	Calado et al., 2012	GEO: GSE39443
Germinal Center signature	Brescia et al., 2018	GEO: GSE110669
Germinal Center Ythdf2-deficient B cells	Grenov et al., 2021	GEO: GSE180359
Single cell RNA seq	This paper	GEO: GSE189819https://singlecell.broadinstitute.org/single_cell/study/SCP1813/scrnaseq-of-control-and-ythdf2-deficient-antigen-specific-b-cells-from-lymph-nodes-of-immunized-mice?tab=scatter#study-visualize
m6A-seq	This paper	GEO: GSE189819
YTHDF2 RIPseq	Zheng et al., 2020	GEO: GSE136419

**Experimental models: Organisms/strains**		

C57BL/6JOlaHsd mouse	Envigo	N/A
Rosa26 TdTomato C57BL/6 transgenic mouse	Jackson Laboratories	RRID: IMSR_JAX:007914
GFP C57BL/6 transgenic mouse	Jackson Laboratories	RRID: IMSR_JAX:004353
CD23cre C57BL/6 transgenic mouse	Prof. M. Busslinger	N/A
Ythdf2HA-GFP-Fl C57BL/6 transgenic mouse	Prof. D. O’Carroll	N/A
B1-8hi and b1-8lo C57BL/6 transgenic mice	Prod.Michel Nussenzweig	RRID: IMSR_JAX:007594
YTHDF1^−/−^ C57BL/6 transgenic mouse	Prof. Jacob Hanna	N/A
YTHDF3^−/−^ C57BL/6 transgenic mouse	Prof. Jacob Hanna	N/A

**Oligonucleotides**		

See Table S1 for qPCR primers	This paper	N/A

**Software and algorithms**		

Seurat (v.4.0.3)	Butler et al., 2018	https://satijalab.org/seurat/
10× Genomics Cell Ranger software v5.0.1	10xGenomics	https://support.10xgenomics.com/
MARS-seq Pipeline	Kohen et al., 2019	N/A
Flowjo 10	Tree Star	https://www.flowjo.com
GSEA	Subramanian et al., 2005; Mootha et al., 2003	https://www.gsea-msigdb.org/gsea/index.jsp
Imaris 9.1.2	Bitplane	https://imaris.oxinst.com/
Prism 9	Graphpad Software	https://www.graphpad.com/

### Resource availability

#### Lead contact

Further information and requests for resources and reagents should be directed to the lead contact, Ziv Shulman (ziv.shulman@weizmann.ac.il).

#### Materials availability

This study did not generate new unique reagents.

### Experimental model and subject details

Complete knockout models of *Ythdf1* and *Ythdf3* were provided by Y. Hanna (Lasman et al., 2020). GFP- and HA-tagged Ythdf2^HA−GFP-Fl^ (DF2^fl/fl^) mice were provided by D. O’Carroll (Ivanova et al., 2017) and crossed to a B cell-specific Cd23-cre strain (CD23-DF2^fl/fl^). The Cd23-cre mice were provided by M. Busslinger (IMP, Vienna). Ythdf1^−/−^, Ythdf3^−/−^ and CD23-DF2^fl/fl^ mice were bred to mice expressing a modified NP-specific B1-8^hi^ B cell receptor as well as a fluorescent marker (GFP or TdTomato). GFP and TdTomato-expressig mice were purchased from Jackson Laboratories. B1-8^hi^ and B1-8^lo^ transgenic mice were a gift from M. Nussenzweig (Rockefeller University, NY). Transgenic mice were maintained in SPF housing conditions and used at 6–18 weeks of age. WT (C57BL/6) mice were purchased from Envigo and used at 6–15 weeks of age. Both male and female mice were used throughout the study and no gender differences were observed. All experiments on mice were approved by the Weizmann Institute Animal Care and Use Committee.

### Method details

#### Adoptive cell transfer

Splenic tissue was passed through a 70μm mesh into 5 mL PBS on ice, and resting B cells were purified using negative-selection anti-CD43 beads and magnetic-activated cell sorting (MACS) (Miltenyi Biotec) according to the manufacturer’s protocol. Igλ^+^ resting B cells were isolated after initial B cell isolation by incubating with 1 μL anti-Igκ-PE antibody for 30min on ice. After washing, Igλ^+^ B cells were enriched by negative selection using anti-PE magnetic beads (Miltenyi Biotec). CD4^+^ T cells were isolated from splenic cells in a similar manner using CD4^+^ T cell isolation kit (Miltenyi Biotec) according to the manufacturer’s instructions. For adoptive transfer, 3–5x10^6^ total B1-8^hi^ B cells, 1 × 10^5^ Igλ^+^ B1-8^hi^ B cells, or 3–4x10^6^ CD4^+^ T cells were transferred by intravenous injection to host mice a minimum of 4 h prior to antigen challenge. In competition experiments, equal numbers of each of the B cell populations were transferred together and the proportions of each cell type were checked by flow cytometry prior to injection.

#### Immunizations

NP-KLH (BioSearch Technologies, CA, USA) was prepared to a final concentration of 0.4 mg/mL in PBS and alum, and 10 μg (25μL) was administered to each footpad. For prime-boost experiments, mice first received a dose of 50 μg OVA prepared in PBS and alum (100μL volume) to the peritoneum. 2–4 weeks after priming, the OVA-specific immune response was boosted by immunizing mice with 25 μg of NP-OVA (adjuvant-free) in each footpad.

#### Flow cytometry

Single-cell suspensions of spleen and lymph nodes were prepared by first washing and then passing each tissue through a 70μm mesh into PBS containing 2% fetal calf serum (FCS) and 1 mM EDTA. After obtaining single-cell suspensions, Fc receptors were blocked by adding (2 μg/mL) anti-16/32 antibody (TruStain FcX, BioLegend) and incubating for 5–10min. For staining of cell surface markers, cells were incubated with a cocktail of fluorescently labeled antibodies for 30 min on ice. Before analysis, samples were washed with 2 mL PBS containing FCS and EDTA. For intracellular staining, surface-labeled cells were fixed and permeabilized using Fixation/Permeabilization Kit (BD Biosciences) according to the manufacturer’s instructions, and then stained for intracellular markers.

Analysis was done on a CytoFLEX (Beckman Coulter) flow cytometer, or in the case of cell sorting, on a FACSAria III Cell Sorter system (Becton Dickinson). Specific populations were gated as follows: GC: live/single, B220^+^ GL-7^+^ CD38^-^ FAS^+^. PCs: live/single, B220^int^ CD138^+^. For cell sorting and subsequent RNA-sequencing, lymph node cell suspensions were stained for markers of undesired cell populations in addition to the positive selection panel (Dump^−^: CD4^-^, CD8^-^, GR-1^-^, F4/80^-^). Cell populations were sorted directly into 40μL Lysis/Binding buffer (Life Technologies), and samples were immediately put on dry ice until further storage at −80°C.

#### Bulk RNA-sequencing and analysis

A modified version of the MARS-seq protocol (Jaitin et al., 2014; Keren-Shaul et al., 2019) to accommodate bulk RNA samples was used to generate RNA sequencing libraries for transcriptional analysis. RNA extraction of sorted cells was performed using the Dynabeads mRNA DIRECT Purification Kit (Invitrogen). Each sample contained 3–5x10^4^ cells, and 12μL of oligo(dT) Dynabeads per sample was used to capture, wash and elute polyA-RNA with 6.5μL of Tris-HCl according to the protocol. Three to four biological replicates were included in each population. For library preparation, each sample was barcoded during reverse transcription and pooled. Pooled samples were cleaned using Agencourt Ampure XP bead cleanup (Beckman Coulter), and underwent second strand synthesis and linear amplification by T7 *in vitro* transcription. Amplified RNA was fragmented to a length of 300bp on average and tagged with Illumina sequences by ligation, followed by reverse transcription and a final round of PCR amplification (11–13 cycles). Libraries were quality checked and quantified by Qubit (Qubit 4 fluorometer, Invitrogen), TapeStation (4150 TapeStation System, Agilent) and qPCR. Sequencing was done using a Nextseq 75 cycle high output kit (Illumina; paired end sequencing).

Alignment and differential expression analysis was performed using the UTAP pipeline (Kohen et al., 2019). Reads were trimmed using Cutadapt and mapped to the Mus_musculus genome (UCSC mm10) using STAR (Dobin et al., 2013) v2.4.2a with default parameters. The pipeline quantifies the genes annotated in RefSeq (extended by 1,000 bases toward the 5′ edge and 100 bases in the 3′ direction). Htseq-count (Anders et al., 2014) (union mode) was used for counting sequenced reads. Expression analysis was based on genes with a minimum of five UMI-corrected reads in at least one sample. Normalization of the counts and differential expression analysis was performed using DESeq2 (Love et al. 2014) with the following parameters: betaPrior = True, cooksCutoff = FALSE, independentFiltering = FALSE. Raw p values were adjusted for multiple testing using the procedure of Benjamini and Hochberg. The GSEA tool (Mootha et al., 2003; Subramanian et al., 2005) was used to analyze changes in specific gene networks. GSEA was run with default parameters.

#### Single-cell RNA-sequencing

Single-cell suspensions from lymph nodes of host mice were independently stained with fluorescently-conjugated antibodies for FACS sorting (see section on flow cytometry) together with 1μg CD45 Cell Hashing antibodies (TotalSeq B anti-mouse CD45, Biolegend) prepared according to the manufacturer’s instructions. Dump^−^ (see “flow cytometry”) transferred B cells were sorted into a cooled 15 mL tube with 0.04% BSA in PBS using a FACS Aria II sorter (BD Bioscience). 20,000 sorted cells were loaded into the 10× Genomics instrument and libraries were created using the Single Cell Expression v3.1 (Dual Index) kit (10× Genomics) according to the manufacturer’s instructions.

#### Single-cell data processing

FASTQ files were aligned to the mouse mm10 reference genome using 10× Genomics Cell Ranger software v5.0.1 to create unique molecular identifier count tables of gene expression for each sample. Unique molecular identifier counts were normalized by library size. The UMI count matrix was converted to Seurat objects using R package Seurat (v.4.0.3). After removing cells with less than 200 genes or more than 10% of UMIs mapped to mitochondrial genes, hashed cells were demultiplexed using HTODemux function, and cells aligned to doublet and negative groups were removed from further analysis. SCTransform function was used to normalize the dataset and Uniform Manifold Approximation and Projection (UMAP) was used to reduce dimensions. For each cell, S phase score and G2 M phase score were estimated with the CellCycleScoring function from Seurat, and these scores were regressed out. Analysis of GO terms enriched in PreGC upregulated genes was done using enrichr (Chen et al., 2013). Differentiation trajectories were fitted with Slingshot package (version 1.6.1) (Street et al., 2018), with the Naive cluster selected as a starting point. Changes in gene expression as a function of pseudotime were visualized using the PlotExpression command.

#### Validation of cell assignment of clusters by gene signature scoring

Signatures of differentially expressed genes between two subpopulations were generated as follows: For follicular B cells, activated B cells, MBCs, and GC B cells, deseq2 results were downloaded from GEO (accession number GSE174394 (Viant et al., 2021)). For splenic B cells compared to follicular B cells, raw counts were downloaded from GEO (accession number GSE60927 (Shi et al., 2015)), and deseq2 was run to calculate log2FC and the adjusted p value for each gene. Each signature consists of genes that are upregulated (FC > 2, padj <0.05) in the indicated cell populations (Figure S1C). The average expression level of each signature was calculated for each cell using the AddModuleScore function.

#### qRT-PCR

Dynabeads mRNA DIRECT™ Purification (Invitrogen) and qScript (Quantabio) kits were used according to the manufacturer’s instructions, to extract poly-A RNA and generate cDNA, respectively. For qPCR reactions, a QuantStudio 5 Real-Time PCR system and Fast SYBR Green Master Mix (Applied Biosystems) were used. Primers are listed in Table S1.

#### *In vitro* proliferation assays

Single-cell suspensions were obtained from mouse spleens and stained with CellTrace-Violet (Molecular Probes) according to the manufacturer’s instructions. Cells were resuspended in RPMI (supplemented with 10% FCS, Penicillin-Streptomycin, L-glutamine, Sodium Pyruvate, β-mercaptoethanol, HEPES, and gentamicin), seeded in a 96 well plate at 1 × 10^6^ cells/mL, and stimulated with αIgM at concentrations ranging from 10 ng to 10 μg per ml for 3 days. Cell proliferation and CD86 expression were analyzed by flow cytometry.

#### Two-photon light microscopy

Intravital imaging was done using a Zeiss LSM 880 upright microscope fitted with a Coherent Chameleon Vision laser. A femtosecond-pulsed two-photon laser tuned to 940 nm was used for image acquisition and the microscope was equipped with a filter cube containing 565 LPXR to split the emission to a photomultiplier tube detector (fitted with a 579–631 nm filter for tdTomato detection). An additional 505 LPXR mirror further split the emission to two GaAsp detectors (with a 500–550 nm filter for GFP detection). Tile images were acquired with Z-stacks of approximately 100 μm, and 5 μm steps between each Z-plane. The zoom was set to 1.5, and pictures were acquired at 512 × 512 × –y resolution. Images were processed using Imaris software (Bitplane). Mean signal intensity was determined using IMARIS surfaces projection tool to identify single cells. Mean GFP fluorescence intensity above background signal (26 arbitrary units, median of YTHDF2-GFP-low cells = 2) was considered YTHDF2-GFP-high expressing cells.

#### M^6^A-seq and analysis of mRNA methylation sites

B cells were isolated from splenic cell suspensions using CD43 magnetic beads and magnetic cell separation (Miltenyi Biotec). Minimum 95% B cell purity was confirmed by flow cytometry analysis. B cells were resuspended in RPMI complete medium (see “in vitro proliferation”) at 1.5 × 10^6^ cells/mL and stimulated with 5 mg/mL LPS for 3 days.

M^6^A immunoprecipitation and RNA-seq library preparation was performed as previously described (Garcia-Campos et al., 2019). Three biological replicates of control and METTL3-deleted samples were included. Reads were aligned to the mouse mm9 genome using STAR aligner. Each site was assigned peak over input (POI) and peak over median (POM) scores representing the fold change of enrichment in the peak region over the corresponding region in the input fraction, or over the median coverage in the gene, respectively. For identification of sites present in the control sample but absent in the METTL3-deleted sample, we performed two separate t-tests to assess whether the POM and/or the POI scores differed significantly (p < 0.05) between control and METTL3-deleted samples. A peak was determined to be METTL3-specific if the mean POI and mean POM scores (across the triplicates) in the WT samples were higher than their counterparts in the METTL3-deleted samples, and if at least one of the two associated p values was significant. For estimating the global methylation levels per sample we calculated POI and POM scores for a pre-assembled set of 16,321 consistently identified m^6^A sites and a set of 30,115 control sites.

#### YTHDF2 RIP-seq data analysis

Fastq files were downloaded from the gene expression omnibus (GSE136419). Reads were aligned to the Mus_musculus genome (UCSC mm10) using STAR (Dobin et al., 2013). Aligned reads were visualized using the Integrated Genomics Viewer (IGV) (Robinson et al., 2011).

#### YTHDF2 RNA immunoprecipitation (RIP)

Isolated B cells from DF2^fl/fl^ and CD23-DF2^fl/fl^ mice were stimulated with 5 μg/mL LPS for three days *in vitro*. Immunoprecipitation (IP) of endogenous RNP complexes from whole-cell extracts was performed as described by (Yoon et al., 2012) with the modification that cell lysates were incubated with antibody prior to incubation with protein A/G beads. In brief, ∼20 M cells per sample were lysed with passive lysis buffer (PLB. 100 mM KCI, 5 mM MgCl2, 10 mM HEPES-NaOH at pH 7, 0.5% NP-40, 1 mM DTT, complete protease inhibitor cocktail (Sigma) and 10u/mL RNAsin (promega)) for 5 min on ice and centrifuged at 20,000 g for 10 min at 4°C. Part of the supernatants was saved as an input sample. The remaining supernatant was incubated in gentle rotation with 5 μg antibody (purified anti-HA.11 epitope Tag antibody, Biolegend, or isotype control) overnight at 4°C with gentle rotation. Protein A/G magnetic beads (GeneScript) were prewashed and incubated with cell lysate + antibody for 4 h at 4°C with gentle rotation. Magnetic beads were washed six times with NT buffer (50 mM Tris-HCL at pH 7.4, 150 mM NaCl, 1 mM MgCl_2_, 0.05% NP-40) and RNA was extracted by TRI reagent (sigma). RNA was converted to cDNA by qScript cDNA synthesis kit (Quantabio) and further analyzed by qPCR. RNA IP levels were normalized to input fraction and compared to Gapdh and IgG isotype controls. The IP fraction from CD23-DF2^fl/fl^ samples had insufficient amounts of RNA for detection by qRT-PCR.

### Quantification and statistical analysis

Statistical significance was determined using Graphpad Prism v.8.0, with the tests used and number of repetitions (n) indicated in each figure legend.

### Additional resources

The scRNA-seq data was deposited in the Single Cell portal and can be accessed through the following link:

https://singlecell.broadinstitute.org/single_cell/study/SCP1813/scrnaseq-of-control-and-ythdf2-deficient-antigen-specific-b-cells-from-lymph-nodes-of-immunized-mice?tab=scatter#study-visualize.

## Data Availability

• This paper analyzes existing, publicly available data. These accession numbers for the datasets are listed in the key resources table. • Single cell RNA-seq, bulk RNA-seq and m6A-seq data have been deposited at GEO and are publicly available as of the date of publication. Accession numbers are listed in the key resources table. • This paper does not report an original code. • Any additional information required to reanalyze the data reported in this paper is available from the lead contact upon request.

## Supplemental information

Document S1. Figures S1 and S2 and Table S1

Document S6. Article plus supplemental information
