# Supplementary material for: YTHDF2 suppresses the plasmablast genetic program and promotes germinal center formation
Source: Cell Rep. 2022 May 6;39(5):110778. doi: 10.1016/j.celrep.2022.110778 (PMC9108551; doi:10.1016/j.celrep.2022.110778)
Supplement: Document S1. Figures S1 and S2 and Table S1 [file mmc1.pdf]

Cell Reports, Volume *39*

Supplemental information

YTHDF2 suppresses the plasmablast genetic program and promotes germinal center formation

Amalie Grenov, Hadas Hezroni, Lior Lasman, Jacob H. Hanna, and Ziv Shulman

Figure S1

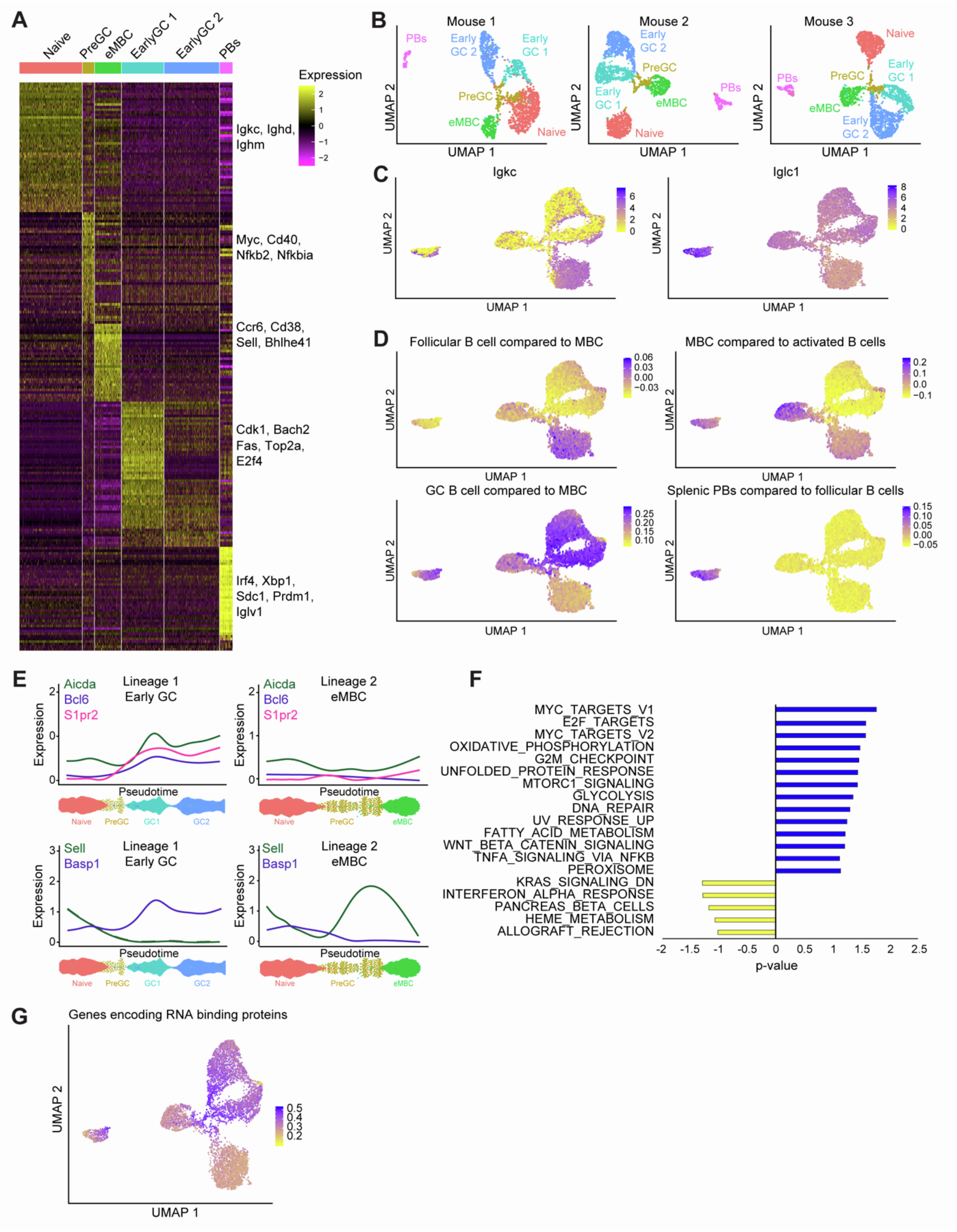
A
Naive
PreGC
eMBC
EarlyGC 1
EarlyGC 2
PBs
Expression
Igkc, Ighd, Ighm
Myc, Cd40, Nfkb2, Nfkbia
Ccr6, Cd38, Sell, Bhlhe41
Cdk1, Bach2 Fas, Top2a, E2f4
Irf4, Xbp1, Sdc1, Prdm1, Iglv1
B
Mouse 1
Mouse 2
Mouse 3
UMAP 1
UMAP 2
C
Igkc
Iglc1
D
Follicular B cell compared to MBC
MBC compared to activated B cells
GC B cell compared to MBC
Splenic PBs compared to follicular B cells
E
Aicda
Bcl6
S1pr2
Lineage 1 Early GC
Lineage 2 eMBC
Sell
Basp1
Expression
Pseudotime
F
MYC_TARGETS_V1
E2F_TARGETS
MYC_TARGETS_V2
OXIDATIVE_PHOSPHORYLATION
G2M_CHECKPOINT
UNFOLDED_PROTEIN_RESPONSE
MTORC1_SIGNALING
GLYCOLYSIS
DNA_REPAIR
UV_RESPONSE_UP
FATTY_ACID_METABOLISM
WNT_BETA_CATENIN_SIGNALING
TNFA_SIGNALING_VIA_NFKB
PEROXISOME
KRAS_SIGNALING_DN
INTERFERON_ALPHA_RESPONSE
PANCREAS_BETA_CELLS
HEME_METABOLISM
ALLOGRAFT_REJECTION
p-value
G
Genes encoding RNA binding proteins

Figure S1. Single cell RNA-seq analysis of antigen specific B cells during the early stages of the adaptive immune response, Related to Figure 1. (**A**) Expression heatmap showing the top 50 upregulated genes for each cluster. (**B**) UMAP projections of scRNA-seq profiles of 1645, 2511, and 2260 transferred B1-8^{hi} B cells isolated from 3 mice, 5 days after immunization. Clusters in the UMAP plots are color-coded according to different cell populations. Annotation of cell populations was done based on the same markers as in Figure 1. (**C, D**) UMAP projections of signature scores. Each signature consists of genes that are upregulated (FC > 2, padj < 0.05) in the indicated cell populations. RNA-seq samples were downloaded from Gene Expression Omnibus accession no. GSE174394 (follicular B cells, activated B cells, MBCs, GC B cells) and GSE60927 (splenic PBs, follicular B cells). (**E**) Expression of *Bcl6*, *Aicda*, *S1pr2*, *Sell* and *Basp1*, along the B cell differentiation trajectories defined in Figure 1I. (**F**) Normalized enrichment scores of hallmark gene sets enrichment analysis of genes whose expression is significantly upregulated in the Pre-GC cluster compared to the Naive and eMBC cell clusters. (**G**) UMAP projections of RBPs gene signature scores.

Figure S2

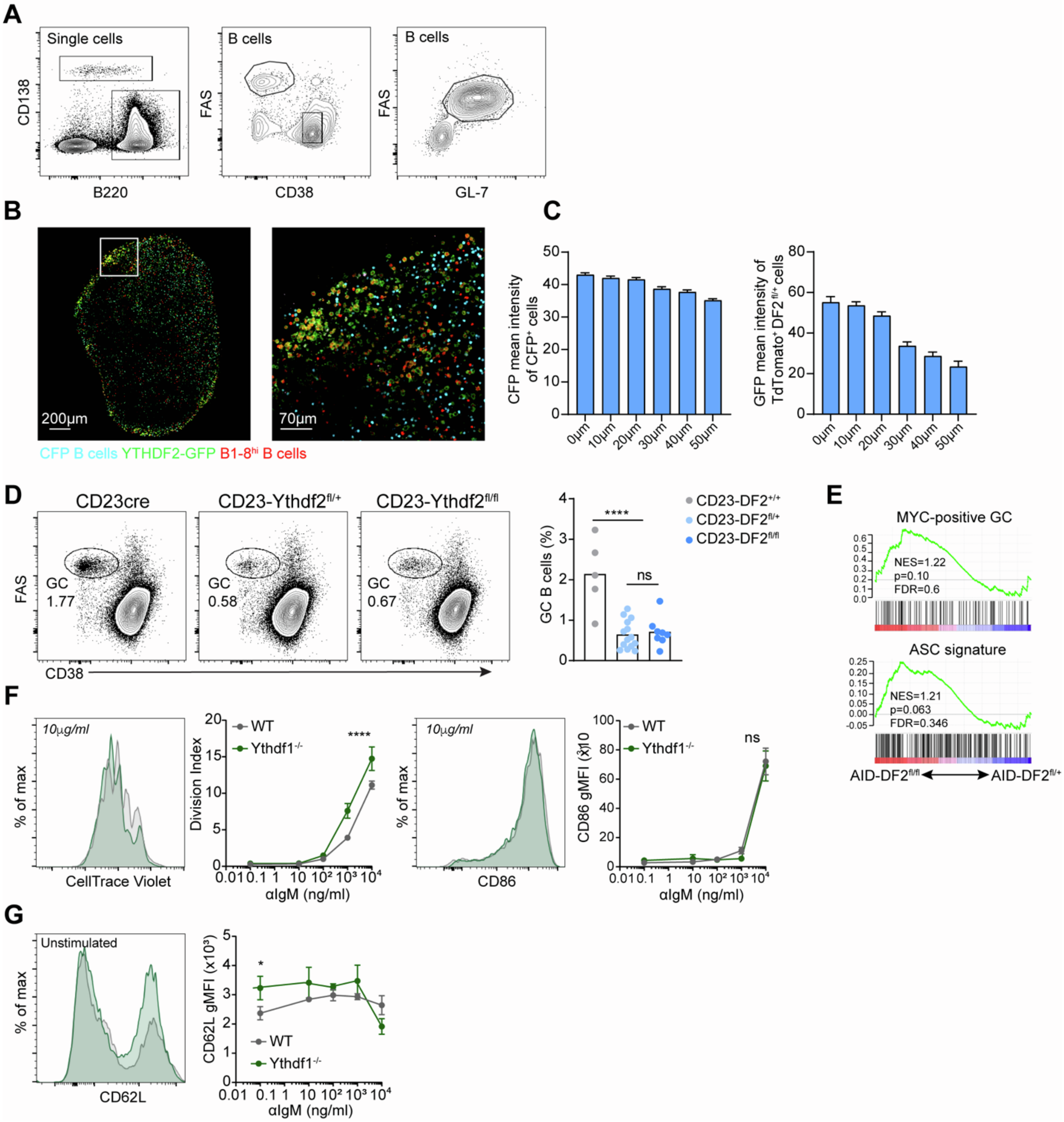
A
Single cells
CD138
B220
B cells
FAS
CD38
B cells
FAS
GL-7
B
200μm
70μm
CFP B cells YTHDF2-GFP B1-8hi B cells
C
CFP mean intensity of CFP+ cells
GFP mean intensity of TdTomato+ DF2fl/+ cells
0μm
10μm
20μm
30μm
40μm
50μm
D
CD23cre
CD23-Ythdf2fl/+
CD23-Ythdf2fl/fl
FAS
CD38
GC 1.77
GC 0.58
GC 0.67
GC B cells (%)
****
ns
CD23-DF2+/+
CD23-DF2fl/+
CD23-DF2fl/fl
E
MYC-positive GC
NES=1.22
p=0.10
FDR=0.6
ASC signature
NES=1.21
p=0.063
FDR=0.346
AID-DF2fl/fl
AID-DF2fl/+
F
10μg/ml
% of max
CellTrace Violet
Division Index
WT
Ythdf1-/-
****
αIgM (ng/ml)
CD86
ns
G
Unstimulated
CD62L
CD62L gMFI (x10³)
*

Figure S2. YTHDF2 and YTHDF1 functions in the B cell immune response, Related to Figure 2, 3 and 6. (A) Gating strategy for plasma cells and B cells (left), GC B cells , Naive B cells (middle), and FAS^{+} $GL\text{-}7^{+}$ B cells (right). **(B-C)** Two-photon imaging of $TdTomato^{+}$ $DF2^{fl/+}$ B cells (transferred prior to immunization) and polyclonal CFP^{+} naive B cells (transferred after immunization) three days after NP-KLH immunization. **(C)** Quantification of CFP and GFP mean fluorescence intensity measured in Z-stacks with 10μm increments from the LN cortex. **(D)** CD23-$DF2^{+/+}$, CD23-$DF2^{fl/+}$, CD23-$DF2^{fl/fl}$ mice were immunized with NP-KLH and analyzed for GC formation after 7 days by flow cytometry. Data were pooled from 5-14 independent mice. **(E)** GSEA analysis of Ythdf2-deficient GC LZ B cells from GSE180359, using gene signatures obtained from MYC-positive GC B cells: GSE39443, ASC: GSE60927. **(F, G)** Splenic cells from Ythdf1-deficient and WT mice were stained with CellTrace Violet and stimulated with increasing concentrations of anti-IgM for three days in vitro. CD86 expression, CellTrace Violet dilution and CD62L expression were assessed by flow cytometry. Plots are representative of three independent experiments. Dots represent mean±SD. Data were pooled from two independent experiments each with three samples per group. Statistical significance was tested using one-way ANOVA followed by Holm-Sidak's multiple comparisons test (A), two-way ANOVA followed by Sidak's multiple comparisons test (B, C), or using two-tailed unpaired Student's t-test. (D). $^{*}P < 0.05$; $^{**}P < 0.01$; $^{****} P < 0.001$. NS, not significant.

Table S1. List of primers for RT-qPCR, Related to Star Methods

**Primer Name**	**Organism**	**Primer sequence (5'-3')**
Ubc_fwd	Mouse	GCCCAGTGTTACCACCAAGA
Ubc_rev	Mouse	CCCATCACACCCAAGAACA
Ythdf1_1_fwd	Mouse	ACAGTTACCCCTCGATGAGTG
Ythdf1_1_rev	Mouse	GGTAGTGAGATACGGGATGGGA
Ythdf1_2_fwd	Mouse	GAGCAGTTACACTTACCCACC
Ythdf1_2_rev	Mouse	TGTTGAGGGAGTCACTGTGAAA
Ythdf1_3_fwd	Mouse	CACAGTGACTCCCTCAACAAG
Ythdf1_3_rev	Mouse	AGGTGGTAACATCCCCAATCTT
Ythdf2_1_fwd	Mouse	GAGCAGAGACCAAAAGGTCAAG
Ythdf2_1_rev	Mouse	CTGTGGGCTCAAGTAAGGTTC
Ythdf2_2_fwd	Mouse	TATGGACAACTGAGCAACGGA
Ythdf2_2_rev	Mouse	GAGCTGGGTGCATAAGCGTA
Ythdf3_1_fwd	Mouse	CGATTCATCAAAAAGATGCTG
Ythdf3_1_rev	Mouse	TCTGACATTGGTGGATAGCTG
Ythdf3_2_fwd	Mouse	CATAGGGCAACAGAGGAAACAG
Ythdf3_2_rev	Mouse	ATCTCCAGCCGTGGACCAT
Sell_PP2_F	Mouse	CCCCTTGGGAAACTTCAGCTT
Sell_PP2_R	Mouse	ACCATGACGGCTACAGGAATG
Ccr7_1_F	Mouse	CAGGTGTGCTTCTGCCAAGAT
Ccr7_1_R	Mouse	GGTAGGTATCCGTCATGGTCT
CXCR5_F	Mouse	ATGAACTACCCACTAACCCTGG

Primer Name	Organism	Primer sequence (5'-3')
CXCR5_R	Mouse	TGTAGGGGAATCTCCGTGCT
Itgal_PP1_F	Mouse	CCAGACTTTTGCTACTGGGAC
Itgal_PP1_R	Mouse	GCTTGTTCGGCAGTGATAGAG
membrane IgM left	Mouse	CCTCTTCTACAGCACCACCG
membrane IgM right	Mouse	TGGTCTCTGCTGTCCTTCCA
secretory IgM left	Mouse	CACACTGTACAATGTCTCCCTGA
secretory IgM right	Mouse	GGTTAGTTTGCATACACAGAGCA
